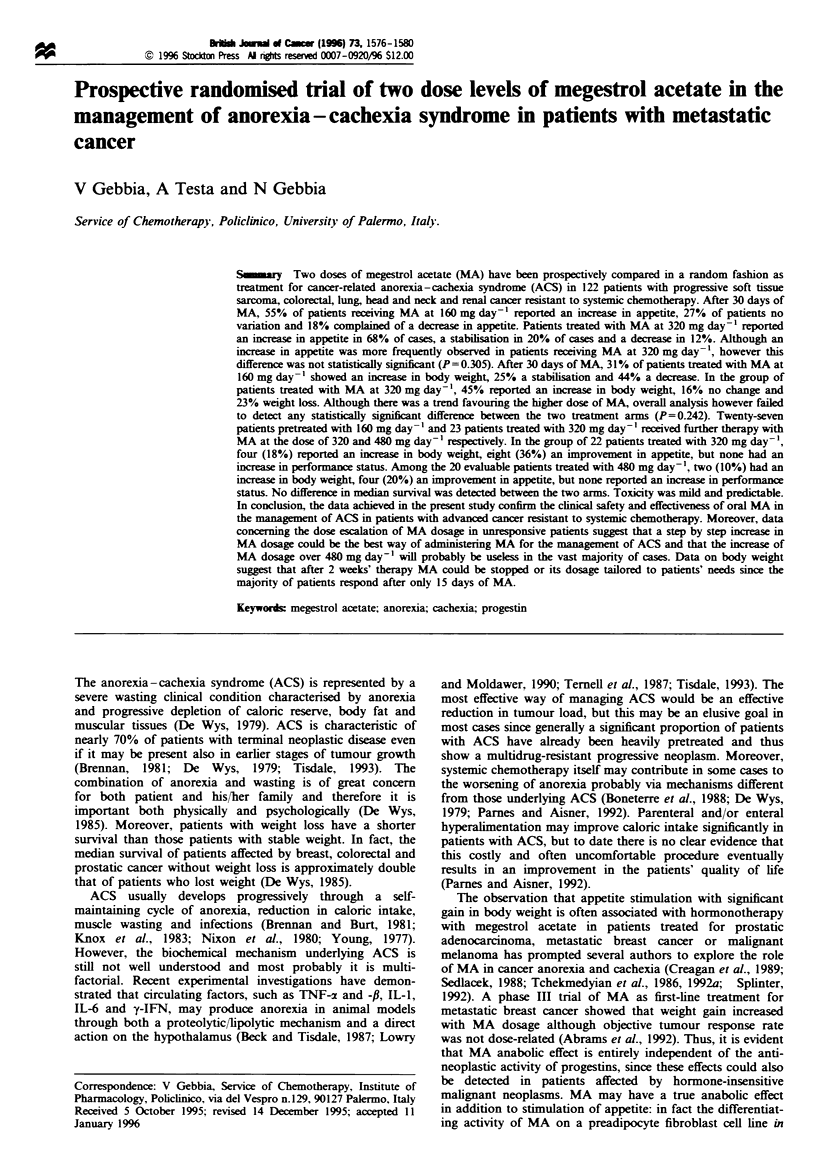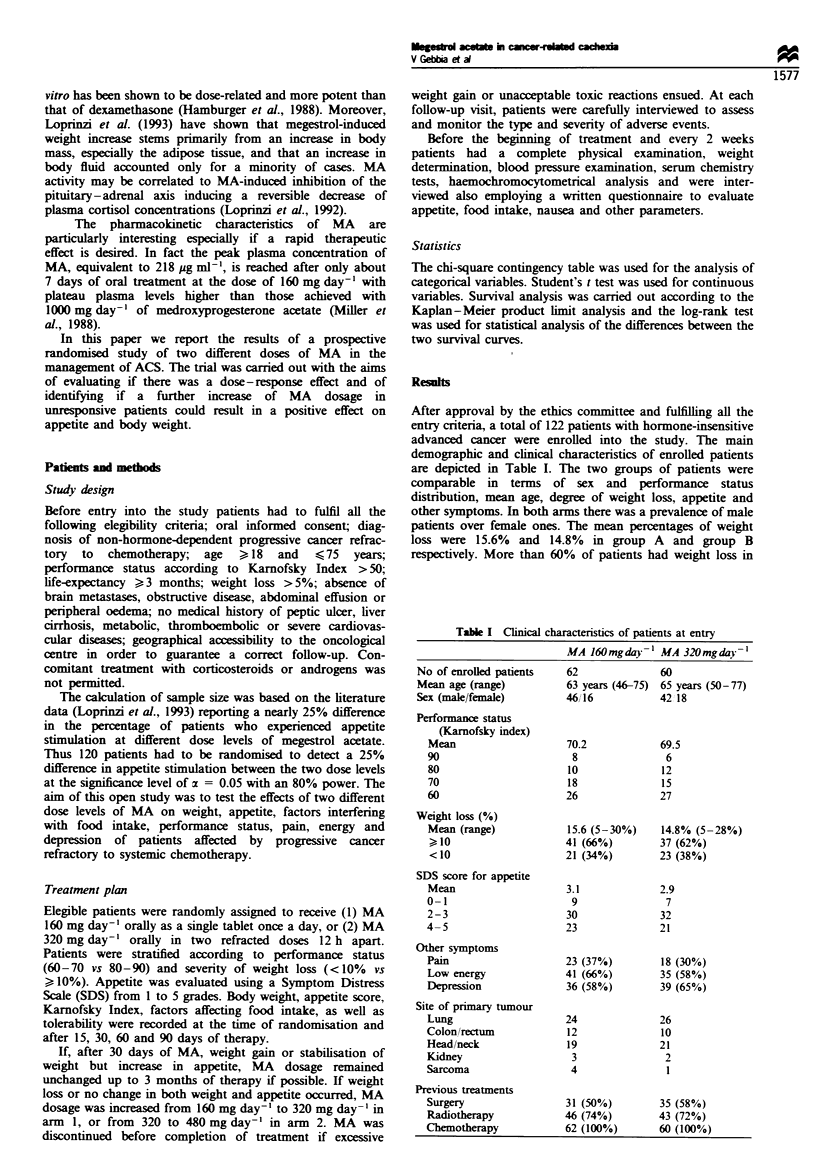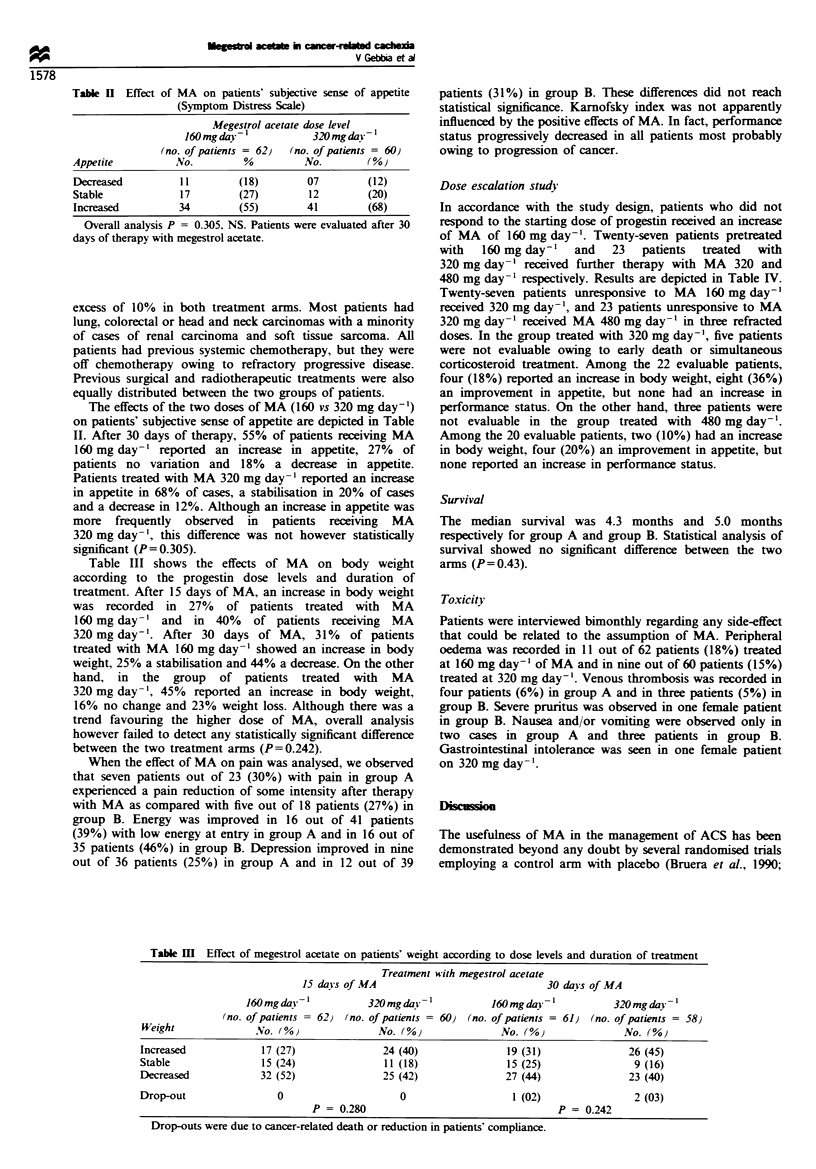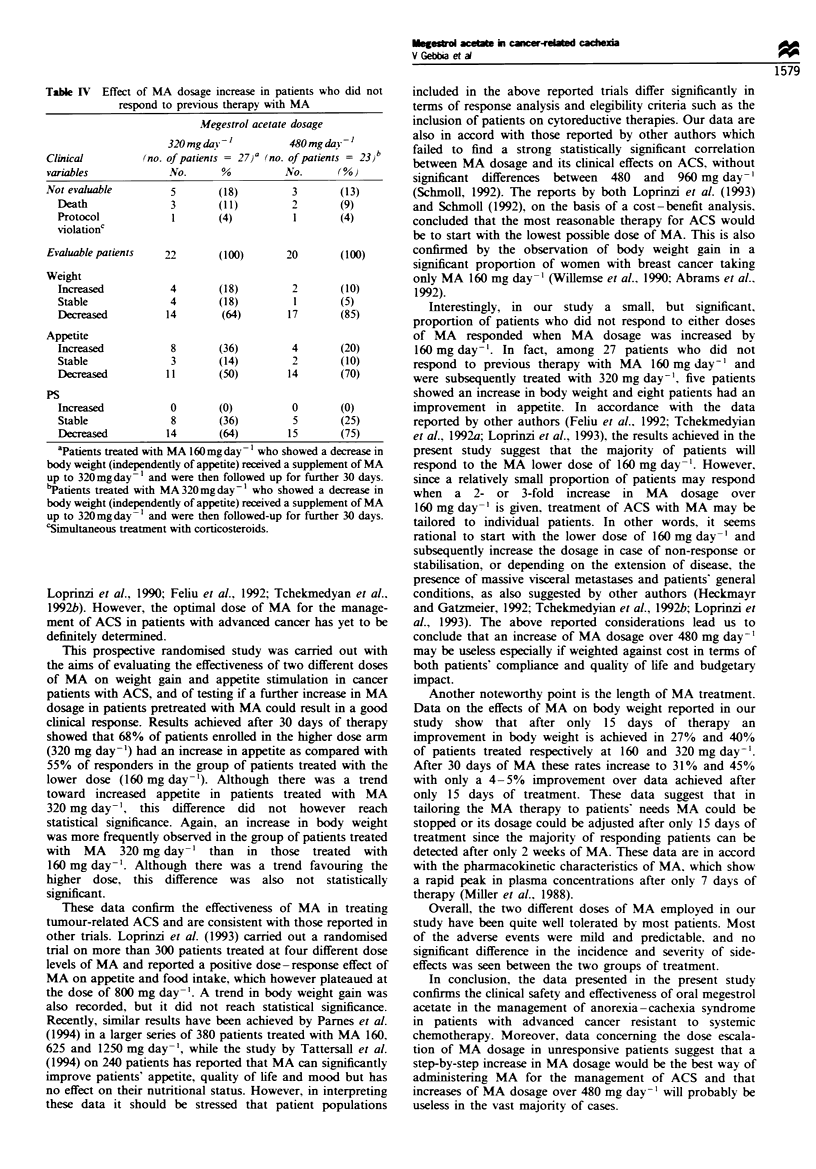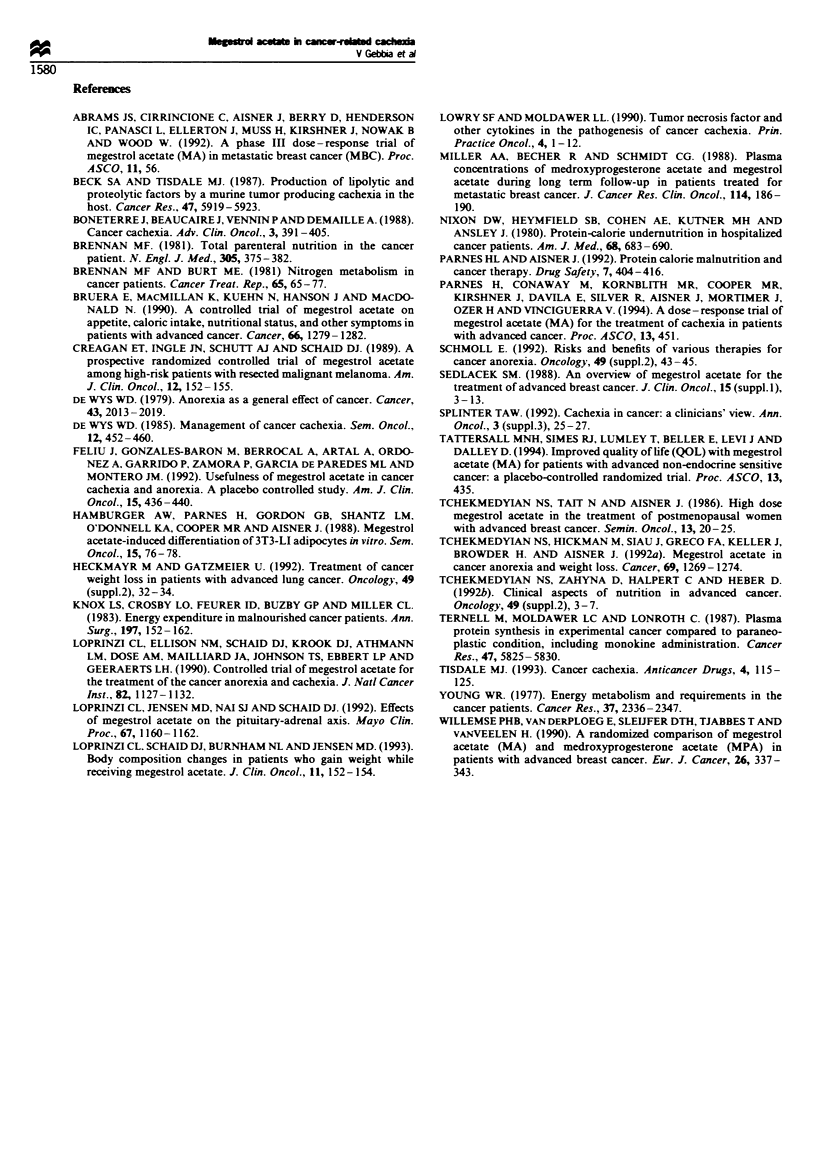# Prospective randomised trial of two dose levels of megestrol acetate in the management of anorexia-cachexia syndrome in patients with metastatic cancer.

**DOI:** 10.1038/bjc.1996.297

**Published:** 1996-06

**Authors:** V. Gebbia, A. Testa, N. Gebbia

**Affiliations:** Service of Chemotherapy, University of Palermo, Italy.

## Abstract

Two doses of megestrol acetate (MA) have been prospectively compared in a random fashion as treatment for cancer-related anorexia-cachexia syndrome (ACS) in 122 patients with progressive soft tissue sarcoma, colorectal, lung, head and neck and renal cancer resistant to systemic chemotherapy. After 30 days of MA, 55% of patients receiving MA at 160 mg day-1 reported an increase in appetite, 27% of patients no variation and 18% complained of a decrease in appetite. Patients treated with MA at 320 mg day-1 reported an increase in appetite in 68% of cases, a stabilisation in 20% of cases and a decrease in 12%. Although an increase in appetite was more frequently observed in patients receiving MA at 320 mg day-1, however this difference was not statistically significant (P = 0.305). After 30 days of MA, 31% of patients treated with MA at 160 mg day-1 showed an increase in body weight, 25% a stabilisation and 44% a decrease. In the group of patients treated with MA at 320 mg day-1, 45% reported an increase in body weight, 16% no change and 23% weight loss. Although there was a trend favouring the higher dose of MA, overall analysis however failed to detect any statistically significant difference between the two treatment arms (P = 0.242). Twenty-seven patients pretreated with 160 mg day-1 and 23 patients treated with 320 mg day-1 received further therapy with MA at the dose of 320 and 480 mg day-1 respectively. In the group of 22 patients treated with 320 mg day-1 four (18%) reported an increase in body weight, eight (36%) an improvement in appetite, but none had an increase in performance status. Among the 20 evaluable patients treated with 480 mg day-1, two (10%) had an increase in body weight, four (20%) an improvement in appetite, but none reported an increase in performance status. No difference in median survival was detected between the two arms. Toxicity was mild and predictable. In conclusion, the data achieved in the present study confirm the clinical safety and effectiveness of oral MA in the management of ACS in patients with advanced cancer resistant to systemic chemotherapy. Moreover, data concerning the dose escalation of MA dosage in unresponsive patients suggest that a step by step increase in MA dosage could be the best way of administering MA for the management of ACS and that the increase of MA dosage over 480 mg day-1 will probably be useless in the vast majority of cases. Data on body weight suggest that after 2 weeks' therapy MA could be stopped or its dosage tailored to patients' needs since the majority of patients respond after only 15 days of MA.


© 1996 Stockton Press All rights reserved 0007-0920/96 $12.00

### Prospective randomised trial of two dose levels of megestrol acetate in the management of anorexia-cachexia syndrome in patients with metastatic cancer

V Gebbia, A Testa and N Gebbia

*Service of Chemotherapy, Policlinico, University of Palermo, Italy.*

**Summary** Two doses of megestrol acetate (MA) have been prospectively compared in a random fashion as treatment for cancer-related anorexia-cachexia syndrome (ACS) in 122 patients with progressive soft tissue sarcoma, colorectal, lung, head and neck and renal cancer resistant to systemic chemotherapy. After 30 days of MA, 55% of patients receiving MA at 160 mg day$^{-1}$ reported an increase in appetite, 27% of patients no variation and 18% complained of a decrease in appetite. Patients treated with MA at 320 mg day$^{-1}$ reported an increase in appetite in 68% of cases, a stabilisation in 20% of cases and a decrease in 12%. Although an increase in appetite was more frequently observed in patients receiving MA at 320 mg day$^{-1}$, however this difference was not statistically significant ($P=0.305$). After 30 days of MA, 31% of patients treated with MA at 160 mg day$^{-1}$ showed an increase in body weight, 25% a stabilisation and 44% a decrease. In the group of patients treated with MA at 320 mg day$^{-1}$, 45% reported an increase in body weight, 16% no change and 23% weight loss. Although there was a trend favouring the higher dose of MA, overall analysis however failed to detect any statistically significant difference between the two treatment arms ($P=0.242$). Twenty-seven patients pretreated with 160 mg day$^{-1}$ and 23 patients treated with 320 mg day$^{-1}$ received further therapy with MA at the dose of 320 and 480 mg day$^{-1}$ respectively. In the group of 22 patients treated with 320 mg day$^{-1}$, four (18%) reported an increase in body weight, eight (36%) an improvement in appetite, but none had an increase in performance status. Among the 20 evaluable patients treated with 480 mg day$^{-1}$, two (10%) had an increase in body weight, four (20%) an improvement in appetite, but none reported an increase in performance status. No difference in median survival was detected between the two arms. Toxicity was mild and predictable. In conclusion, the data achieved in the present study confirm the clinical safety and effectiveness of oral MA in the management of ACS in patients with advanced cancer resistant to systemic chemotherapy. Moreover, data concerning the dose escalation of MA dosage in unresponsive patients suggest that a step by step increase in MA dosage could be the best way of administering MA for the management of ACS and that the increase of MA dosage over 480 mg day$^{-1}$ will probably be useless in the vast majority of cases. Data on body weight suggest that after 2 weeks' therapy MA could be stopped or its dosage tailored to patients' needs since the majority of patients respond after only 15 days of MA.

**Keywords:** megestrol acetate; anorexia; cachexia; progestin

The anorexia-cachexia syndrome (ACS) is represented by a severe wasting clinical condition characterised by anorexia and progressive depletion of caloric reserve, body fat and muscular tissues (De Wys, 1979). ACS is characteristic of nearly 70% of patients with terminal neoplastic disease even if it may be present also in earlier stages of tumour growth (Brennan, 1981; De Wys, 1979; Tisdale, 1993). The combination of anorexia and wasting is of great concern for both patient and his/her family and therefore it is important both physically and psychologically (De Wys, 1985). Moreover, patients with weight loss have a shorter survival than those patients with stable weight. In fact, the median survival of patients affected by breast, colorectal and prostatic cancer without weight loss is approximately double that of patients who lost weight (De Wys, 1985).

ACS usually develops progressively through a self-maintaining cycle of anorexia, reduction in caloric intake, muscle wasting and infections (Brennan and Burt, 1981; Knox *et al.*, 1983; Nixon *et al.*, 1980; Young, 1977). However, the biochemical mechanism underlying ACS is still not well understood and most probably it is multifactorial. Recent experimental investigations have demonstrated that circulating factors, such as TNF-$\alpha$ and -$\beta$, IL-1, IL-6 and $\gamma$-IFN, may produce anorexia in animal models through both a proteolytic/lipolytic mechanism and a direct action on the hypothalamus (Beck and Tisdale, 1987; Lowry and Moldawer, 1990; Ternell *et al.*, 1987; Tisdale, 1993). The most effective way of managing ACS would be an effective reduction in tumour load, but this may be an elusive goal in most cases since generally a significant proportion of patients with ACS have already been heavily pretreated and thus show a multidrug-resistant progressive neoplasm. Moreover, systemic chemotherapy itself may contribute in some cases to the worsening of anorexia probably via mechanisms different from those underlying ACS (Boneterre *et al.*, 1988; De Wys, 1979; Parnes and Aisner, 1992). Parenteral and/or enteral hyperalimentation may improve caloric intake significantly in patients with ACS, but to date there is no clear evidence that this costly and often uncomfortable procedure eventually results in an improvement in the patients' quality of life (Parnes and Aisner, 1992).

The observation that appetite stimulation with significant gain in body weight is often associated with hormonotherapy with megestrol acetate in patients treated for prostatic adenocarcinoma, metastatic breast cancer or malignant melanoma has prompted several authors to explore the role of MA in cancer anorexia and cachexia (Creagan *et al.*, 1989; Sedlacek, 1988; Tchekmedyian *et al.*, 1986, 1992*a*; Splinter, 1992). A phase III trial of MA as first-line treatment for metastatic breast cancer showed that weight gain increased with MA dosage although objective tumour response rate was not dose-related (Abrams *et al.*, 1992). Thus, it is evident that MA anabolic effect is entirely independent of the antineoplastic activity of progestins, since these effects could also be detected in patients affected by hormone-insensitive malignant neoplasms. MA may have a true anabolic effect in addition to stimulation of appetite: in fact the differentiating activity of MA on a preadipocyte fibroblast cell line *in*

Correspondence: V Gebbia, Service of Chemotherapy, Institute of Pharmacology, Policlinico, via del Vespro n.129, 90127 Palermo, Italy
Received 5 October 1995; revised 14 December 1995; accepted 11 January 1996

*vitro* has been shown to be dose-related and more potent than that of dexamethasone (Hamburger *et al.*, 1988). Moreover, Loprinzi *et al.* (1993) have shown that megestrol-induced weight increase stems primarily from an increase in body mass, especially the adipose tissue, and that an increase in body fluid accounted only for a minority of cases. MA activity may be correlated to MA-induced inhibition of the pituitary-adrenal axis inducing a reversible decrease of plasma cortisol concentrations (Loprinzi *et al.*, 1992).

The pharmacokinetic characteristics of MA are particularly interesting especially if a rapid therapeutic effect is desired. In fact the peak plasma concentration of MA, equivalent to 218 $\mu g\ ml^{-1}$, is reached after only about 7 days of oral treatment at the dose of 160 mg $day^{-1}$ with plateau plasma levels higher than those achieved with 1000 mg $day^{-1}$ of medroxyprogesterone acetate (Miller *et al.*, 1988).

In this paper we report the results of a prospective randomised study of two different doses of MA in the management of ACS. The trial was carried out with the aims of evaluating if there was a dose-response effect and of identifying if a further increase of MA dosage in unresponsive patients could result in a positive effect on appetite and body weight.

### Patients and methods

### *Study design*

Before entry into the study patients had to fulfil all the following elegibility criteria; oral informed consent; diagnosis of non-hormone-dependent progressive cancer refractory to chemotherapy; age $\geqslant 18$ and $\leqslant 75$ years; performance status according to Karnofsky Index $>50$; life-expectancy $\geqslant 3$ months; weight loss $>5\%$; absence of brain metastases, obstructive disease, abdominal effusion or peripheral oedema; no medical history of peptic ulcer, liver cirrhosis, metabolic, thromboembolic or severe cardiovascular diseases; geographical accessibility to the oncological centre in order to guarantee a correct follow-up. Concomitant treatment with corticosteroids or androgens was not permitted.

The calculation of sample size was based on the literature data (Loprinzi *et al.*, 1993) reporting a nearly 25% difference in the percentage of patients who experienced appetite stimulation at different dose levels of megestrol acetate. Thus 120 patients had to be randomised to detect a 25% difference in appetite stimulation between the two dose levels at the significance level of $\alpha = 0.05$ with an 80% power. The aim of this open study was to test the effects of two different dose levels of MA on weight, appetite, factors interfering with food intake, performance status, pain, energy and depression of patients affected by progressive cancer refractory to systemic chemotherapy.

### *Treatment plan*

Elegible patients were randomly assigned to receive (1) MA 160 mg $day^{-1}$ orally as a single tablet once a day, or (2) MA 320 mg $day^{-1}$ orally in two refracted doses 12 h apart. Patients were stratified according to performance status (60-70 *vs* 80-90) and severity of weight loss ($<10\%$ *vs* $\geqslant 10\%$). Appetite was evaluated using a Symptom Distress Scale (SDS) from 1 to 5 grades. Body weight, appetite score, Karnofsky Index, factors affecting food intake, as well as tolerability were recorded at the time of randomisation and after 15, 30, 60 and 90 days of therapy.

If, after 30 days of MA, weight gain or stabilisation of weight but increase in appetite, MA dosage remained unchanged up to 3 months of therapy if possible. If weight loss or no change in both weight and appetite occurred, MA dosage was increased from 160 mg $day^{-1}$ to 320 mg $day^{-1}$ in arm 1, or from 320 to 480 mg $day^{-1}$ in arm 2. MA was discontinued before completion of treatment if excessive weight gain or unacceptable toxic reactions ensued. At each follow-up visit, patients were carefully interviewed to assess and monitor the type and severity of adverse events.

Before the beginning of treatment and every 2 weeks patients had a complete physical examination, weight determination, blood pressure examination, serum chemistry tests, haemochromocytometrical analysis and were interviewed also employing a written questionnaire to evaluate appetite, food intake, nausea and other parameters.

### *Statistics*

The chi-square contingency table was used for the analysis of categorical variables. Student's *t* test was used for continuous variables. Survival analysis was carried out according to the Kaplan-Meier product limit analysis and the log-rank test was used for statistical analysis of the differences between the two survival curves.

### Results

After approval by the ethics committee and fulfilling all the entry criteria, a total of 122 patients with hormone-insensitive advanced cancer were enrolled into the study. The main demographic and clinical characteristics of enrolled patients are depicted in Table I. The two groups of patients were comparable in terms of sex and performance status distribution, mean age, degree of weight loss, appetite and other symptoms. In both arms there was a prevalence of male patients over female ones. The mean percentages of weight loss were 15.6% and 14.8% in group A and group B respectively. More than 60% of patients had weight loss in

**Table I** Clinical characteristics of patients at entry

| | *MA 160 mg* $day^{-1}$ | *MA 320 mg* $day^{-1}$ |
|---|---|---|
| No of enrolled patients | 62 | 60 |
| Mean age (range) | 63 years (46-75) | 65 years (50-77) |
| Sex (male/female) | 46/16 | 42/18 |
| Performance status (Karnofsky index) | | |
| Mean | 70.2 | 69.5 |
| 90 | 8 | 6 |
| 80 | 10 | 12 |
| 70 | 18 | 15 |
| 60 | 26 | 27 |
| Weight loss (%) | | |
| Mean (range) | 15.6 (5-30%) | 14.8% (5-28%) |
| $\geqslant 10$ | 41 (66%) | 37 (62%) |
| $<10$ | 21 (34%) | 23 (38%) |
| SDS score for appetite | | |
| Mean | 3.1 | 2.9 |
| 0-1 | 9 | 7 |
| 2-3 | 30 | 32 |
| 4-5 | 23 | 21 |
| Other symptoms | | |
| Pain | 23 (37%) | 18 (30%) |
| Low energy | 41 (66%) | 35 (58%) |
| Depression | 36 (58%) | 39 (65%) |
| Site of primary tumour | | |
| Lung | 24 | 26 |
| Colon/rectum | 12 | 10 |
| Head/neck | 19 | 21 |
| Kidney | 3 | 2 |
| Sarcoma | 4 | 1 |
| Previous treatments | | |
| Surgery | 31 (50%) | 35 (58%) |
| Radiotherapy | 46 (74%) | 43 (72%) |
| Chemotherapy | 62 (100%) | 60 (100%) |

**Table II** Effect of MA on patients' subjective sense of appetite (Symptom Distress Scale)

| Appetite | Megestrol acetate dose level 160 mg day$^{-1}$ (no. of patients = 62) No. | % | 320 mg day$^{-1}$ (no. of patients = 60) No. | (%) |
|---|---|---|---|---|
| Decreased | 11 | (18) | 07 | (12) |
| Stable | 17 | (27) | 12 | (20) |
| Increased | 34 | (55) | 41 | (68) |

Overall analysis $P$ = 0.305, NS. Patients were evaluated after 30 days of therapy with megestrol acetate.

excess of 10% in both treatment arms. Most patients had lung, colorectal or head and neck carcinomas with a minority of cases of renal carcinoma and soft tissue sarcoma. All patients had previous systemic chemotherapy, but they were off chemotherapy owing to refractory progressive disease. Previous surgical and radiotherapeutic treatments were also equally distributed between the two groups of patients.

The effects of the two doses of MA (160 *vs* 320 mg day$^{-1}$) on patients' subjective sense of appetite are depicted in Table II. After 30 days of therapy, 55% of patients receiving MA 160 mg day$^{-1}$ reported an increase in appetite, 27% of patients no variation and 18% a decrease in appetite. Patients treated with MA 320 mg day$^{-1}$ reported an increase in appetite in 68% of cases, a stabilisation in 20% of cases and a decrease in 12%. Although an increase in appetite was more frequently observed in patients receiving MA 320 mg day$^{-1}$, this difference was not however statistically significant ($P$=0.305).

Table III shows the effects of MA on body weight according to the progestin dose levels and duration of treatment. After 15 days of MA, an increase in body weight was recorded in 27% of patients treated with MA 160 mg day$^{-1}$ and in 40% of patients receiving MA 320 mg day$^{-1}$. After 30 days of MA, 31% of patients treated with MA 160 mg day$^{-1}$ showed an increase in body weight, 25% a stabilisation and 44% a decrease. On the other hand, in the group of patients treated with MA 320 mg day$^{-1}$, 45% reported an increase in body weight, 16% no change and 23% weight loss. Although there was a trend favouring the higher dose of MA, overall analysis however failed to detect any statistically significant difference between the two treatment arms ($P$=0.242).

When the effect of MA on pain was analysed, we observed that seven patients out of 23 (30%) with pain in group A experienced a pain reduction of some intensity after therapy with MA as compared with five out of 18 patients (27%) in group B. Energy was improved in 16 out of 41 patients (39%) with low energy at entry in group A and in 16 out of 35 patients (46%) in group B. Depression improved in nine out of 36 patients (25%) in group A and in 12 out of 39 patients (31%) in group B. These differences did not reach statistical significance. Karnofsky index was not apparently influenced by the positive effects of MA. In fact, performance status progressively decreased in all patients most probably owing to progression of cancer.

### *Dose escalation study*

In accordance with the study design, patients who did not respond to the starting dose of progestin received an increase of MA of 160 mg day$^{-1}$. Twenty-seven patients pretreated with 160 mg day$^{-1}$ and 23 patients treated with 320 mg day$^{-1}$ received further therapy with MA 320 and 480 mg day$^{-1}$ respectively. Results are depicted in Table IV. Twenty-seven patients unresponsive to MA 160 mg day$^{-1}$ received 320 mg day$^{-1}$, and 23 patients unresponsive to MA 320 mg day$^{-1}$ received MA 480 mg day$^{-1}$ in three refracted doses. In the group treated with 320 mg day$^{-1}$, five patients were not evaluable owing to early death or simultaneous corticosteroid treatment. Among the 22 evaluable patients, four (18%) reported an increase in body weight, eight (36%) an improvement in appetite, but none had an increase in performance status. On the other hand, three patients were not evaluable in the group treated with 480 mg day$^{-1}$. Among the 20 evaluable patients, two (10%) had an increase in body weight, four (20%) an improvement in appetite, but none reported an increase in performance status.

### *Survival*

The median survival was 4.3 months and 5.0 months respectively for group A and group B. Statistical analysis of survival showed no significant difference between the two arms ($P$=0.43).

### *Toxicity*

Patients were interviewed bimonthly regarding any side-effect that could be related to the assumption of MA. Peripheral oedema was recorded in 11 out of 62 patients (18%) treated at 160 mg day$^{-1}$ of MA and in nine out of 60 patients (15%) treated at 320 mg day$^{-1}$. Venous thrombosis was recorded in four patients (6%) in group A and in three patients (5%) in group B. Severe pruritus was observed in one female patient in group B. Nausea and/or vomiting were observed only in two cases in group A and three patients in group B. Gastrointestinal intolerance was seen in one female patient on 320 mg day$^{-1}$.

### Discussion

The usefulness of MA in the management of ACS has been demonstrated beyond any doubt by several randomised trials employing a control arm with placebo (Bruera *et al*., 1990;

**Table III** Effect of megestrol acetate on patients' weight according to dose levels and duration of treatment

| Weight | Treatment with megestrol acetate: 15 days of MA, 160 mg day$^{-1}$ (no. of patients = 62) No. (%) | 15 days of MA, 320 mg day$^{-1}$ (no. of patients = 60) No. (%) | 30 days of MA, 160 mg day$^{-1}$ (no. of patients = 61) No. (%) | 30 days of MA, 320 mg day$^{-1}$ (no. of patients = 58) No. (%) |
|---|---|---|---|---|
| Increased | 17 (27) | 24 (40) | 19 (31) | 26 (45) |
| Stable | 15 (24) | 11 (18) | 15 (25) | 9 (16) |
| Decreased | 32 (52) | 25 (42) | 27 (44) | 23 (40) |
| Drop-out | 0 | 0 | 1 (02) | 2 (03) |
| | $P$ = 0.280 | | $P$ = 0.242 | |

Drop-outs were due to cancer-related death or reduction in patients' compliance.

Table IV Effect of MA dosage increase in patients who did not respond to previous therapy with MA

| Clinical variables | Megestrol acetate dosage 320 mg day$^{-1}$ (no. of patients = 27)[a] No. | % | 480 mg day$^{-1}$ (no. of patients = 23)[b] No. | (%) |
|---|---|---|---|---|
| *Not evaluable* | 5 | (18) | 3 | (13) |
| Death | 3 | (11) | 2 | (9) |
| Protocol violation[c] | 1 | (4) | 1 | (4) |
| *Evaluable patients* | 22 | (100) | 20 | (100) |
| Weight | | | | |
| Increased | 4 | (18) | 2 | (10) |
| Stable | 4 | (18) | 1 | (5) |
| Decreased | 14 | (64) | 17 | (85) |
| Appetite | | | | |
| Increased | 8 | (36) | 4 | (20) |
| Stable | 3 | (14) | 2 | (10) |
| Decreased | 11 | (50) | 14 | (70) |
| PS | | | | |
| Increased | 0 | (0) | 0 | (0) |
| Stable | 8 | (36) | 5 | (25) |
| Decreased | 14 | (64) | 15 | (75) |

[a]Patients treated with MA 160 mg day$^{-1}$ who showed a decrease in body weight (independently of appetite) received a supplement of MA up to 320 mg day$^{-1}$ and were then followed up for further 30 days. [b]Patients treated with MA 320 mg day$^{-1}$ who showed a decrease in body weight (independently of appetite) received a supplement of MA up to 320 mg day$^{-1}$ and were then followed-up for further 30 days. [c]Simultaneous treatment with corticosteroids.

Loprinzi *et al.*, 1990; Feliu *et al.*, 1992; Tchekmedyan *et al.*, 1992*b*). However, the optimal dose of MA for the management of ACS in patients with advanced cancer has yet to be definitely determined.

This prospective randomised study was carried out with the aims of evaluating the effectiveness of two different doses of MA on weight gain and appetite stimulation in cancer patients with ACS, and of testing if a further increase in MA dosage in patients pretreated with MA could result in a good clinical response. Results achieved after 30 days of therapy showed that 68% of patients enrolled in the higher dose arm (320 mg day$^{-1}$) had an increase in appetite as compared with 55% of responders in the group of patients treated with the lower dose (160 mg day$^{-1}$). Although there was a trend toward increased appetite in patients treated with MA 320 mg day$^{-1}$, this difference did not however reach statistical significance. Again, an increase in body weight was more frequently observed in the group of patients treated with MA 320 mg day$^{-1}$ than in those treated with 160 mg day$^{-1}$. Although there was a trend favouring the higher dose, this difference was also not statistically significant.

These data confirm the effectiveness of MA in treating tumour-related ACS and are consistent with those reported in other trials. Loprinzi *et al.* (1993) carried out a randomised trial on more than 300 patients treated at four different dose levels of MA and reported a positive dose–response effect of MA on appetite and food intake, which however plateaued at the dose of 800 mg day$^{-1}$. A trend in body weight gain was also recorded, but it did not reach statistical significance. Recently, similar results have been achieved by Parnes *et al.* (1994) in a larger series of 380 patients treated with MA 160, 625 and 1250 mg day$^{-1}$, while the study by Tattersall *et al.* (1994) on 240 patients has reported that MA can significantly improve patients' appetite, quality of life and mood but has no effect on their nutritional status. However, in interpreting these data it should be stressed that patient populations included in the above reported trials differ significantly in terms of response analysis and elegibility criteria such as the inclusion of patients on cytoreductive therapies. Our data are also in accord with those reported by other authors which failed to find a strong statistically significant correlation between MA dosage and its clinical effects on ACS, without significant differences between 480 and 960 mg day$^{-1}$ (Schmoll, 1992). The reports by both Loprinzi *et al.* (1993) and Schmoll (1992), on the basis of a cost–benefit analysis, concluded that the most reasonable therapy for ACS would be to start with the lowest possible dose of MA. This is also confirmed by the observation of body weight gain in a significant proportion of women with breast cancer taking only MA 160 mg day$^{-1}$ (Willemse *et al.*, 1990; Abrams *et al.*, 1992).

Interestingly, in our study a small, but significant, proportion of patients who did not respond to either doses of MA responded when MA dosage was increased by 160 mg day$^{-1}$. In fact, among 27 patients who did not respond to previous therapy with MA 160 mg day$^{-1}$ and were subsequently treated with 320 mg day$^{-1}$, five patients showed an increase in body weight and eight patients had an improvement in appetite. In accordance with the data reported by other authors (Feliu *et al.*, 1992; Tchekmedyian *et al.*, 1992*a*; Loprinzi *et al.*, 1993), the results achieved in the present study suggest that the majority of patients will respond to the MA lower dose of 160 mg day$^{-1}$. However, since a relatively small proportion of patients may respond when a 2- or 3-fold increase in MA dosage over 160 mg day$^{-1}$ is given, treatment of ACS with MA may be tailored to individual patients. In other words, it seems rational to start with the lower dose of 160 mg day$^{-1}$ and subsequently increase the dosage in case of non-response or stabilisation, or depending on the extension of disease, the presence of massive visceral metastases and patients' general conditions, as also suggested by other authors (Heckmayr and Gatzmeier, 1992; Tchekmedyian *et al.*, 1992*b*; Loprinzi *et al.*, 1993). The above reported considerations lead us to conclude that an increase of MA dosage over 480 mg day$^{-1}$ may be useless especially if weighted against cost in terms of both patients' compliance and quality of life and budgetary impact.

Another noteworthy point is the length of MA treatment. Data on the effects of MA on body weight reported in our study show that after only 15 days of therapy an improvement in body weight is achieved in 27% and 40% of patients treated respectively at 160 and 320 mg day$^{-1}$. After 30 days of MA these rates increase to 31% and 45% with only a 4–5% improvement over data achieved after only 15 days of treatment. These data suggest that in tailoring the MA therapy to patients' needs MA could be stopped or its dosage could be adjusted after only 15 days of treatment since the majority of responding patients can be detected after only 2 weeks of MA. These data are in accord with the pharmacokinetic characteristics of MA, which show a rapid peak in plasma concentrations after only 7 days of therapy (Miller *et al.*, 1988).

Overall, the two different doses of MA employed in our study have been quite well tolerated by most patients. Most of the adverse events were mild and predictable, and no significant difference in the incidence and severity of side-effects was seen between the two groups of treatment.

In conclusion, the data presented in the present study confirms the clinical safety and effectiveness of oral megestrol acetate in the management of anorexia–cachexia syndrome in patients with advanced cancer resistant to systemic chemotherapy. Moreover, data concerning the dose escalation of MA dosage in unresponsive patients suggest that a step-by-step increase in MA dosage would be the best way of administering MA for the management of ACS and that increases of MA dosage over 480 mg day$^{-1}$ will probably be useless in the vast majority of cases.

### References

ABRAMS JS, CIRRINCIONE C, AISNER J, BERRY D, HENDERSON IC, PANASCI L, ELLERTON J, MUSS H, KIRSHNER J, NOWAK B AND WOOD W. (1992). A phase III dose–response trial of megestrol acetate (MA) in metastatic breast cancer (MBC). *Proc. ASCO*, **11**, 56.

BECK SA AND TISDALE MJ. (1987). Production of lipolytic and proteolytic factors by a murine tumor producing cachexia in the host. *Cancer Res.*, **47**, 5919–5923.

BONETERRE J, BEAUCAIRE J, VENNIN P AND DEMAILLE A. (1988). Cancer cachexia. *Adv. Clin. Oncol.*, **3**, 391–405.

BRENNAN MF. (1981). Total parenteral nutrition in the cancer patient. *N. Engl. J. Med.*, **305**, 375–382.

BRENNAN MF AND BURT ME. (1981) Nitrogen metabolism in cancer patients. *Cancer Treat. Rep.*, **65**, 65–77.

BRUERA E, MACMILLAN K, KUEHN N, HANSON J AND MACDONALD N. (1990). A controlled trial of megestrol acetate on appetite, caloric intake, nutritional status, and other symptoms in patients with advanced cancer. *Cancer*, **66**, 1279–1282.

CREAGAN ET, INGLE JN, SCHUTT AJ AND SCHAID DJ. (1989). A prospective randomized controlled trial of megestrol acetate among high-risk patients with resected malignant melanoma. *Am. J. Clin. Oncol.*, **12**, 152–155.

DE WYS WD. (1979). Anorexia as a general effect of cancer. *Cancer*, **43**, 2013–2019.

DE WYS WD. (1985). Management of cancer cachexia. *Sem. Oncol.*, **12**, 452–460.

FELIU J, GONZALES-BARON M, BERROCAL A, ARTAL A, ORDONEZ A, GARRIDO P, ZAMORA P, GARCIA DE PAREDES ML AND MONTERO JM. (1992). Usefulness of megestrol acetate in cancer cachexia and anorexia. A placebo controlled study. *Am. J. Clin. Oncol.*, **15**, 436–440.

HAMBURGER AW, PARNES H, GORDON GB, SHANTZ LM, O'DONNELL KA, COOPER MR AND AISNER J. (1988). Megestrol acetate-induced differentiation of 3T3-LI adipocytes *in vitro*. *Sem. Oncol.*, **15**, 76–78.

HECKMAYR M AND GATZMEIER U. (1992). Treatment of cancer weight loss in patients with advanced lung cancer. *Oncology*, **49** (suppl.2), 32–34.

KNOX LS, CROSBY LO, FEURER ID, BUZBY GP AND MILLER CL. (1983). Energy expenditure in malnourished cancer patients. *Ann. Surg.*, **197**, 152–162.

LOPRINZI CL, ELLISON NM, SCHAID DJ, KROOK DJ, ATHMANN LM, DOSE AM, MAILLIARD JA, JOHNSON TS, EBBERT LP AND GEERAERTS LH. (1990). Controlled trial of megestrol acetate for the treatment of the cancer anorexia and cachexia. *J. Natl Cancer Inst.*, **82**, 1127–1132.

LOPRINZI CL, JENSEN MD, NAI SJ AND SCHAID DJ. (1992). Effects of megestrol acetate on the pituitary-adrenal axis. *Mayo Clin. Proc.*, **67**, 1160–1162.

LOPRINZI CL, SCHAID DJ, BURNHAM NL AND JENSEN MD. (1993). Body composition changes in patients who gain weight while receiving megestrol acetate. *J. Clin. Oncol.*, **11**, 152–154.

LOWRY SF AND MOLDAWER LL. (1990). Tumor necrosis factor and other cytokines in the pathogenesis of cancer cachexia. *Prin. Practice Oncol.*, **4**, 1–12.

MILLER AA, BECHER R AND SCHMIDT CG. (1988). Plasma concentrations of medroxyprogesterone acetate and megestrol acetate during long term follow-up in patients treated for metastatic breast cancer. *J. Cancer Res. Clin. Oncol.*, **114**, 186–190.

NIXON DW, HEYMFIELD SB, COHEN AE, KUTNER MH AND ANSLEY J. (1980). Protein-calorie undernutrition in hospitalized cancer patients. *Am. J. Med.*, **68**, 683–690.

PARNES HL AND AISNER J. (1992). Protein calorie malnutrition and cancer therapy. *Drug Safety*, **7**, 404–416.

PARNES H, CONAWAY M, KORNBLITH MR, COOPER MR, KIRSHNER J, DAVILA E, SILVER R, AISNER J, MORTIMER J, OZER H AND VINCIGUERRA V. (1994). A dose–response trial of megestrol acetate (MA) for the treatment of cachexia in patients with advanced cancer. *Proc. ASCO*, **13**, 451.

SCHMOLL E. (1992). Risks and benefits of various therapies for cancer anorexia. *Oncology*, **49** (suppl.2), 43–45.

SEDLACEK SM. (1988). An overview of megestrol acetate for the treatment of advanced breast cancer. *J. Clin. Oncol.*, **15** (suppl.1), 3–13.

SPLINTER TAW. (1992). Cachexia in cancer: a clinicians' view. *Ann. Oncol.*, **3** (suppl.3), 25–27.

TATTERSALL MNH, SIMES RJ, LUMLEY T, BELLER E, LEVI J AND DALLEY D. (1994). Improved quality of life (QOL) with megestrol acetate (MA) for patients with advanced non-endocrine sensitive cancer: a placebo-controlled randomized trial. *Proc. ASCO*, **13**, 435.

TCHEKMEDYIAN NS, TAIT N AND AISNER J. (1986). High dose megestrol acetate in the treatment of postmenopausal women with advanced breast cancer. *Semin. Oncol.*, **13**, 20–25.

TCHEKMEDYIAN NS, HICKMAN M, SIAU J, GRECO FA, KELLER J, BROWDER H. AND AISNER J. (1992*a*). Megestrol acetate in cancer anorexia and weight loss. *Cancer*, **69**, 1269–1274.

TCHEKMEDYIAN NS, ZAHYNA D, HALPERT C AND HEBER D. (1992*b*). Clinical aspects of nutrition in advanced cancer. *Oncology*, **49** (suppl.2), 3–7.

TERNELL M, MOLDAWER LC AND LONROTH C. (1987). Plasma protein synthesis in experimental cancer compared to paraneoplastic condition, including monokine administration. *Cancer Res.*, **47**, 5825–5830.

TISDALE MJ. (1993). Cancer cachexia. *Anticancer Drugs*, **4**, 115–125.

YOUNG WR. (1977). Energy metabolism and requirements in the cancer patients. *Cancer Res.*, **37**, 2336–2347.

WILLEMSE PHB, VAN DERPLOEG E, SLEIJFER DTH, TJABBES T AND VANVEELEN H. (1990). A randomized comparison of megestrol acetate (MA) and medroxyprogesterone acetate (MPA) in patients with advanced breast cancer. *Eur. J. Cancer*, **26**, 337–343.